# The General Nature and Treatment of Tumours

**Published:** 1845-07

**Authors:** 


### Art. X.

*The General Nature and Treatment of Tumours.* By George Macilwain, Fellow of the Royal College of Surgeons of England, &c. &c.—*London,* 1845. 8vo, pp. 220.

Mr. Macilwain states in his preface the object and plan of his work. He has endeavoured to *sketch* the principles and the application of what he calls "Organic Surgery," to the treatment of tumours. The plan pursued, he observes, is very simple. He has endeavoured to give:—1. A general idea of the nature of tumours. 2. To impress on the practitioner's mind that they must be the result of the actions of one or more of the various organs, on the materials subjected to them. 3. That if the result be unhealthy, the action in a practical sense must be unhealthy also. 4. That in discovering the organ or organs at fault, we must be careful to distinguish health from disease. 5. That, to ascertain the causes of the latter, we must enlarge our investigation. 6. He has suggested the manner; adding "hints" on the treatment of different organs. Lastly. He has offered, as illustrations, a *few* of the results he has already obtained.

We will begin with the practical results of the treatment, and then notice the theories upon which it is founded. The tendency of the plan of treatment in the words of the author, as a whole, is to improve the general condition of the body, and therefore if it procure not the absorption of a tumour, it can by no perceptible possibility do mischief. It proposes no new system, it acts on no new system, it rests on no new hypothesis, it takes the facts as they exist already, &c.; all which we are bound to say is most true. The book is simply a laboured attempt to give an air of novelty to "the principles I imbibed from Mr. Abernethy." In the following case of a lady, Mr. Macilwain shall speak for himself, and as there was no "one function in her whole body going rightly, except the kidney," it may be considered as the fairest possible example of his plan of treatment.

"A lady, ætat. 39, consulted me for a tumour of the breast, under circumstances that were as hopeless as it is possible to imagine; and it is a very good case, so far as showing what may be done where there is organic disease.

"She had had the tumour for some years; and I found, in my notes of the first conversation with me, that she had consulted four surgeons, three of them men of great eminence in London; besides some physicians; and that they had—some more, some less strongly—urged its removal. To this she would not consent. On examining her breast, I found that which was described in my notes at the time, as 'true specimen of carcinoma,' very hard, and adherent to the subjacent parts. The skin is tucked in at the nipple, and a dark spot there, slightly abraded. It is painful, with a 'sense of drawing.' Arm swollen, and red in the vicinity of the tumour. There issues from the dark spot a few drops of blood daily, which began when she made use of, as she believes, some irritating application.

"In this lady I could not find any one function in her whole body going rightly, except the kidney, and that was only from her own report; and yet I could find nothing unusual in her habits, except that from about the age of 20, previously to which she had been very active, her habits had become sedentary, catamenia were scanty and pale, and never lasting more than a day; liver extremely torpid, skin chilly, bowels habitually costive, appetite deficient. She had a number of other symptoms, such as frequent palpitations; but, as she called on me, I did not examine the condition of the abdomen until I visited her, when I found the liver hard and enlarged. Her tongue was very peculiar; I never remember to have seen any like it in a living person; it was shrivelled and pale, covered with a remarkably thick coating of something that I never saw before, but not unlike the tongue that you sometimes see after death in advanced stages of putrefaction. She knew perfectly well that there was no chance of cure; but as she suffered greatly, and seemed willing to do anything that was recommended, I did everything I could to persuade her to be careful; assuring her that, although we did not allow ourselves to talk of curing such diseases as that she laboured under, yet they were far from being beyond the control of treatment altogether; that a careful mode of life, adapted to the peculiar fault in the organs, was often rewarded by an almost entire immunity from pain, and a stationary condition of the tumour; and that I should not despair of accomplishing something in her case; not, said I, so much from any *peculiarity* of plan, as that I cannot glean from you that any very particular attention has at any time been paid to your diet and mode of life, by which alone your organs can be got into better condition. Well, we set to work, and before she had been three weeks on the plan, she said that the tumour had not been so easy for twelve preceding months. For the treatment, so far as I directed it, consisted of very plain diet, the rigid exclusion of sugar, and grease of all kinds—friction to the skin—with especial avoidance generally of any in the neighbourhood of the tumour; when the catamenia occurred, such as they were, a few leeches were ordered to be applied to the pubes on their cessation. Her medicines were chiefly aloes and ipecacuanha, with now and then three grains of calomel, so guarded as not to act too quickly; but aloes and ipecacuanha were the medicines generally used. Now this case went on until the tumour had become decidedly loose, that is, more moveable, and the suffering from it very trivial; it was now and then a little uncomfortable, but she was generally *easy*. Although she herself referred her uneasy moments to slight aberrations from the plan, still she could not help occasionally transgressing; and an unfortunate consultation served only to render matters worse, in the following manner." (pp. 194-7.)

And then Mr. Macilwain states how an "eminent physician," called into consultation, told the patient she might eat what she pleased, and how the lady, acting on this opinion, was shortly "thrown into a state of terrible suffering," which opium failed to relieve, and which was only ameliorated by a return to the former plan. This poor daughter of Eve could not, however, with all this bitter experience, avoid tasting Mr. Macilwain's forbidden foods. "She frequently tampered with impunity; but once too often," as he pathetically assures us, "and the result was what I have stated—she died in great agony." It was not the awful cancer, but (as Mr. Macilwain's rigid induction proves) the "tampering," that slew her.

The next case is that of a very hard tumour in the lip.

"A lady, about 34 years of age, had a very hard tumour in the centre and occupying about one third of the upper lip. It had a very firm and well-defined boundary, was of a circular form, and, though deeply imbedded in the substance of the part, was very moveable. The lower surface of the tumour was denuded of its integuments, presenting an excoriated rather than an ulcerated surface. She suffered considerable pain occasionally, and the denuded surface was exquisitely sensitive. Some months had elapsed since her attention had been first excited to it, and the tumour had gradually acquired its present characters. She appeared much out of health; a bilious, dull, leaden complexion was accompanied by deficient appetite, irregular and painful menstruation, torpid bowels, cold skin, pain in the head, &c. Having carefully examined the case, I told her that I feared nothing could be of any service but the removal of the disease, and that I perfectly concurred in the advice given by Mr. Kingdon, viz. that so soon as her health was somewhat improved to allow him to remove it. Mr. Kingdon wished me to take an analytical account of her case, and try whether it were possible, by any measure, to influence the condition of the tumour. I, therefore, took down her case in the tabular form which I recommended; and, on a careful review of the history and present phenomena, was led to regard the liver and uterus as the organs *primarily* and chiefly affected. Not to enter unnecessarily into details, I may briefly state that the organs to which my endeavours were directed were the liver, skin, and uterus. Her diet was simple and strictly defined, and she kept memoranda of the various articles of diet she employed, together with such other matters as I recommended, after the plan of which I have already spoken. The lip was to be kept still; she was to speak as little as possible, and to take her food through the spout of a teapot. She was allowed to put a bread-and-water poultice to the lip at night, *when the tumour was painful*, and to defend it from the atmosphere in the day time by a little spermaceti ointment, applied warm, by means of a camel-hair brush. The medicines she took were aloes, antimony, or ipecacuanha, and confection of opium, in different modifications and doses, according to her condition, and now and then, but rarely, a single dose of calomel and confection of opium. Besides these, until the skin became more tractable (as I wished the kidney to be more liberal) I gave her nitrate of potash with sarsaparilla. She was to take daily exercise, and to have her skin well rubbed. She, also, in the course of the treatment, had a tartar emetic plaster applied to the pubes, which appeared an useful auxiliary in restoring a more healthy condition of the catamenia. The whole treatment lasted six months. At first her looks began to improve, then her functions to become more regular, and at length alterations were observed in the tumour, first a softening, and subsequently a diminution of its bulk. The absorption continued slowly but progressively, until the whole tumour had disappeared." (pp. 201-3.)

Mr. Macilwain recites other and similarly successful cases, and observes generally that he knows no form of [morbid] deposition, to which the mode of treatment he advocates has been fairly applied which has not been influenced by it. Bronchocele, and "depositions of whose site there could be doubt as seated in the liver" have been thus influenced, the former when iodine had failed.

The local applications are classed under two heads: 1, "such as profess to excite actions in the part, and 2, such as are supposed to act by excluding such injurious influences from without as might impede or embarrass the salutary operations of nature." In the former Mr. Macilwain has little confidence: the judicious management of the latter is he thinks very important in most cases of tumour. He regards "extreme cleanliness, from use of tepid and sometimes cold water, with the careful defence of any accessible surface from acrid secretions by the interposition of spermaceti ointment, or any other mild oleaginous matter, as representing the principal benefits derivable from local measures."

We must now notice Mr. Macilwain's hypothetical notions.

According to Mr. Macilwain's idea, tumours "are not swellings, but consist of newly-deposited matter (in or on a part) which is in some sense or other unnatural. . . . . All unnatural depositions result from the disturbance of the function of some organ or organs of the animal economy, and are to be treated by the correction of that function." It is now satisfactorily ascertained that hydatid tumours consist of parasitical animals, and of the morbid deposits they excite; these animals being not formed within the body, but entering into it from without as microscopic ova or otherwise, and in several instances making the circuit of the circulating system. We here more particularly refer to Klencke's researches. If these be correct, the inapplicability of Mr. Macilwain's hypothesis as a general principle is manifest. The histological anatomy of scirrhus and other forms of tumour is equally opposed to this doctrine, which is, in fact, the old notion of metastatic deposit tricked out by meager references to modern researches with which Mr. Macilwain is evidently but imperfectly acquainted, and with laments on "the hardship under which men labour who have little but their enthusiasm to cheer them on through the, alas, too lonely path of inductive inquiry."

According to Mr. Macilwain, it is indisputable that tumours represent some function, and "that inquiry into the nature of a tumour should always recognize the possibility of a relation existing between the chemical elements of the tumour, and an imperfect performance of some function dealing with such elements." The absurdity of this as applied to hydatid tumours is manifest. Mr. Macilwain, however, acknowledges that he has "not yet arrived at the ascertainment of such relation in the chemical sense," but he "believes" he is "approaching that point," and has "the highest hopes of still better things;" all which faith and hope evidently form a large proportion of Mr. Macilwain's inductive philosophy; unless there be among his "bulky manuscripts" a history of numerous chemical analyses.

The hypothetical doctrines of Mr. Macilwain necessarily lead him to such considerations as his limited ideas and knowledge will permit, of the various functions of organs, and their absolute and relative importance to the health in general and to tumours in particular. That he may be able more distinctly to note changes in function he adopts a formula of investigation, which he terms "a Table of Record," and which we doubt not would be found useful in practice if carefully used. The great difficulty in the use of such formulæ (more elaborate than that of Mr. Macilwain are to be found in French and German authors) is that they are only suited to clinical instruction. It is seldom that practitioners can spare the time necessary for the required investigations, and as seldom will the patient afford at once the time and the facilities for the manipulation of his person such a method demands for its completion. It is not sufficient that a mode of practice be *good;* it must also be practicable: the union of these two qualities are essential to success. Nothing is so easy as to lay down methods, nothing so difficult as to act up to them.

Mr. Macilwain proceeds to review the functions of the more important viscera. His hints on the management of the different organs are useful and practical; his dietetics, without presenting any novelty, are interesting, and show that, however fantastic his theories may be, he has the good sense and judgment to adapt them to a rational empiricism; of course there are no chemical analyses of the morbid secretions. We subjoin his own summary of his functional or organic dietetics.

"The following, then, are the chief points I desire to impress in the management of the stomach: 1. Moderation. 2. The exclusion of unnecessary variety at a single meal, or (during investigation) in a single day. 3. A careful adjustment of animal or farinaceous food *to the case.* 4. Extension of surface of the food by minute comminution or otherwise extreme division. 5. Avoidance of *all* interfering influences, whether indulgence in condiments or questionable habits, as snuffing, smoking, &c. 6. Determined exercise on foot, horseback, or by gestation. 7. The avoidance of hasty generalization; and until your plan is tolerably defined, desiring your patient to keep a book of diet; in order that you may test your selection, or that of your patient, in detail." (p. 109.)

These directions are excellent; and we believe that many practitioners would greatly improve their practice by keeping them in view; yet we hope that no well-educated physician or surgeon, of even moderate experience, would think that it required any particular stretch of "inductive philosophy" to educe such self-evident propositions: yet so it is, according to Mr. Macilwain.

Our author observes, with reference to the liver, that "no organ is nearly so silent or insidious in its conduct of disease," meaning, we suppose, that there may be extensive disorganization of its structure or derangement of its functions with few or slight symptoms. We take in an immense quantity of "charcoal," and it must go out again. The liver is almost wholly engaged in excreting "charcoal" from the system (at this conclusion Mr. Macilwain arrives by a Macilwainean induction), and "therefore, in the regulation of disordered liver," we quote the inductive philosopher, "I try all I can to diminish the quantity of charcoal, except such as is contained in *necessary* food. A man may eat meat without fat; butter is unnecessary; so is sugar," &c. In all this there seems to us naught novel. Has it not been known from time immemorial that sweets, spirits, and such articles of diet made people bilious?—not exactly, we grant, in the "charcoal" mode, but in some mode, upon the exact nature of which even Liebig would be too modest peremptorily to decide.

We would shortly observe that the inductive philosophy of the other organs of the body is very similar to the preceding. The lungs are "refrigerating organs," and it is by means of them, in combination with the skin, that animals maintain their characteristic temperature, by throwing off superfluous caloric. Mr. Maugham, "a man of striking talent, and a bold and original thinker," first drew the attention of our author "to the subject in the following manner: 'I wish,' said he, 'you would consider whether we do not maintain our temperature by the skin throwing off superfluous heat!' or words to that effect." Mr. Macilwain reminds us of Mrs. Gamp. "Mrs. Harris often and often says to me, 'Sairey Gamp,' she says, 'you really do amaze me,' "*or words to that effect*"—Mrs. Gamp might have added. We cannot really be serious, for the experiments our philosopher details are a burlesque on experimental philosophy. Punch, we are certain, might make something clever out of the experiment on the refrigerating "frogs, salamanders, and water-beetles." A Member of Parliament fresh from a vote on the Maynooth question, "temperature 70° Fahrenheit," would make an admirable refrigerating subject.

Mr. Macilwain's book is a striking illustration of a mind o'ervaulting itself. Ambitious to be a philosopher, he goes about it and about it, ever attempting to rise, but never mounting,—like a man on a treadmill. He is manifestly a pains-taking, persevering, industrious practitioner, and very trustworthy as such: indeed we believe him to be an excellent and successful physician, and we must think well of his general system of practice, as it is one we have ourselves long followed in the treatment of all chronic diseases. Had he, in his writings, confined his expositions to practical hints, and made no pretence to philosophy, we would, we are sure, have had a more pleasant task in dwelling only on his merits as an author. But we are bound to deny all honorable mention of his philosophy; and we cannot entirely pass by without notice some of his other defects. He is ignorant of chemistry, while he criticises Liebig: but then "he has reason to believe that Mr. Maugham, who is certainly eminently qualified for the task, is willing to devote himself to chemical analysis on such terms as will," &c. &c. He has boldly propounded a theory on the formation of tumours "entirely from his own practice," being all the while (by his own confession) "not aware what assistance we are likely to derive from microscopic inquiries." His style, also, although ambitious, is far from accurate, as will be seen from our quotations. Nevertheless, we recommend his book to all our younger readers, as likely to supply them with practical hints of great value in the treatment not only of tumours but of chronic diseases generally, whether surgical or medical. Two great temptations and dangers constantly beset the path of young men educated in this country, when called on to treat chronic maladies,—the temptation and the danger of Heroical Practice and of Empirical Practice. The perusal of Mr. Macilwain's book (if they will study its practical doctrines and let its philosophy pass) is well calculated to suggest to them that more comprehensive, cautious, regimenal, hygienic, safe, and, if we may so speak, natural practice—to which future experience is sure to lead them, and which is alone suited to such cases. If they are led to adopt this early in their career, they will assuredly thereby acquire much immediate satisfaction, and eschew the regret which, sooner or later, must ensue from following an opposite course.

